# Engaging Older Adults in Technology Research: Perspectives on Barriers and Facilitators Using a Case Study Approach

**DOI:** 10.1093/geroni/igaa057.2992

**Published:** 2020-12-16

**Authors:** Susan Kirkland

**Affiliations:** Dalhousie University, Halifax, Nova Scotia, Canada

## Abstract

The aim of OA-INVOLVE is to provide recommendations to support older adult engagement in research that leads to the successful development of technologies for and with older adults. To support this aim we conducted a longitudinal case study project in which we interviewed eight AGE-WELL research teams conducting technology projects to explore the benefits, challenges, and solutions for meaningful engagement. Members of the OA-INVOLVE Older Adult Research Partner Group (OARPG) were involved in all aspects of the project. Findings from the case studies provided important insights regarding structural, contextual, and individual factors that enable and constrain active involvement of older adults. Many projects reported developing unique “workarounds” in order to move beyond involving older adults as participants to involving them as advisors and decision makers. Researchers identified that they often lack the skills, training and resources to engage older adults in a meaningful way and could benefit from capacity building.

